# Editorial: Advancements in biogenic nanoparticles: sustainable solutions for biotechnology

**DOI:** 10.3389/fbioe.2026.2005001

**Published:** 2026-10-06

**Authors:** Silvana Alborés, Paulina L. Paez, Ljubica Tasic

**Affiliations:** 1 Área de Microbiología, Departamento de Biociencias, Facultad de Química, Universidad de la República, Montevideo, Uruguay; 2 Departamento de Ciencias Farmacéuticas, Facultad de Ciencias Químicas, Universidad Nacional de Córdoba, Unidad de Investigación y Desarrollo en Tecnología Farmacéutica (UNITEFA-CONICET), Córdoba, Argentina; 3 Laboratório de Química Biológica, Instituto de Química, Universidade Estadual de Campinas, Campinas, Brazil

**Keywords:** antimicrobial, biogenic nanoparticles, biotechnology, nanotechnology, sustainable solutions

## 1 Introduction

The escalating global crisis of antimicrobial resistance (AMR) represents one of the most formidable threats to modern medicine and public health, raising the prospect of a post-antibiotic era. As conventional small-molecule antibiotics increasingly fail against multidrug-resistant (MDR) priority pathogens, biogenic nanoparticles (BNPs) have emerged as promising, eco-friendly, and highly versatile nanoplatforms (Estevez et al., 2021). Synthesized via biological entities, including plants, bacteria, fungi, algae, and genetically engineered nanocompartments, BNPs provide a sustainable alternative to traditional chemical and physical synthesis routes. By leveraging natural cellular machinery and biological metabolites, green synthesis not only eliminates toxic chemical reductants and harsh reaction conditions but also imparts an intrinsic biomolecular capping layer that governs particle stability, surface charge, biocompatibility, and targeted nano–bio interactions.

This Frontiers Research Topic brings together original research and critical reviews at the intersection of green nanotechnology, biomedical engineering, structural biology, and translational microbiology. The Research Topic highlights the dual role of BNPs: serving as potent, multi-target antimicrobial agents that can circumvent established resistance mechanisms, and as sophisticated, genetically encoded platforms for cellular tracking and diagnostic bioimaging.

### 1.1 Mechanistic advances in green synthesis and antimicrobial performance

The core objective of this Research Topic is to elucidate how biologically mediated synthesis routes dictate the physicochemical architecture and functional performance of BNPs. As Abdallah et al. critically review, green synthesis uses diverse biological “factories” to drive metal ion reduction, nucleation, growth, and natural capping. Phytochemicals (phenolics, flavonoids, terpenoids), microbial enzymes (reductases, hydrogenases), and exopolysaccharides may act individually or synergistically to reduce metal precursors into elemental nanostructures. Falletti et al. report an integrated cascade valorization strategy for *Flaveria bidentis* biomass, utilizing ultrasound-assisted aqueous extraction to yield phenolic-rich extracts that serve directly as reducing and stabilizing agents for the eco-friendly synthesis of antibacterial silver nanoparticles. Highlighting sustainable nanotechnology and biomass valorization, this work demonstrates the potential to transform the invasive species *F. bidentis* into functional, bioactive silver nanoparticles. Ahmad et al. developed a sustainable strategy to produce docosahexaenoic acid (DHA; 22:6 n-3)- loaded silver nanoparticles (AgNPs) using DHA extracted and purified from the marine microalga *Aurantiochytrium limacinum*. The incorporation of DHA modified the morphology and crystallinity of the AgNPs, as confirmed by FTIR analysis, indicating effective interaction between DHA and the nanoparticle surface. The resulting DHA-AgNPs showed enhanced structural stability and multifunctional potential, combining the antimicrobial properties of silver with the biomedical benefits of DHA.

The intrinsic biological capping environment confers enhanced colloidal stability, prevents unwanted agglomeration, and establishes a multi-target mode of action that fundamentally differs from conventional antibiotics:

- Membrane Disruption and Permeabilization: Electrostatic attraction between BNPs and bacterial cell wall components (e.g., teichoic acids in Gram-positive bacteria or lipopolysaccharides in Gram-negative bacteria) causes localized envelope stress, mechanical deformation, and intracellular leakage.
- Reactive Oxygen Species (ROS) Generation: Metal oxide and metallic BNPs induce localized oxidative stress, leading to non-specific lipid peroxidation, protein denaturation, and genomic DNA fragmentation.
- Anti-Biofilm and Anti-Virulence Strategies: Beyond direct bactericidal killing, BNPs inhibit quorum-sensing (QS) signaling systems, downregulate QS-regulated virulence genes (such as pyocyanin production), and disrupt the extracellular polymeric substance (EPS) matrix to penetrate deep within mature bacterial biofilms.

Because these physical, chemical, and metabolic insults occur simultaneously, BNPs could reduce the likelihood of rapid resistance development compared with agents acting through a single molecular target. Nevertheless, the long-term evolution of resistance or tolerance to BNPs requires further investigation.

### 1.2 Bio-nanoparticles for advanced bioimaging and tumor tracking

Expanding the frontier of biogenic nanomaterials beyond plant and microbial extracts, Milovanova et al. present a novel application of genetically encoded protein nanocompartments (encapsulins) as biogenic magnetic platforms. Encapsulins from the thermophilic bacterium *Quasibacillus thermotolerans* (Qt) self-assemble into hollow, 42-nm icosahedral protein shells that function as biological nanoreactors. Incorporating a ferritin-like cargo protein with ferroxidase activity, these genetically engineered shells sequester divalent iron and form ∼25 nm biogenic magnetic nanoparticles inside the protein cage.

By transducing rat C6 glioma cells with genes encoding Qt nanocompartments and an optical reporter, Milovanova et al. established a dual-labeled glioblastoma model. Using the highly sensitive Nonlinear Magnetization/Magnetic Particle Quantification (MPQ) technique—which detects combinatorial frequencies in an alternating magnetic field—the authors systematically evaluated the temporal dynamics of magnetic signal retention in rapidly proliferating tumor cells. This study defines important operational limitations for cell-labeling strategies: while encapsulin-based magnetic reporters are exceptionally suited for non-dividing or slowly dividing cells (such as stem cells or macrophages), their application in rapidly growing solid tumors requires continuous iron loading strategies.

### 1.3 Addressing current bottlenecks and future horizons

Despite the promising advances highlighted in this Research Topic, the translation of biogenic nanoparticles from laboratory scale to industrial and clinical applications requires overcoming several persistent challenges (Abdallah et al.):

- Batch-to-Batch Reproducibility and Standardization: Natural variability in biological source materials (e.g., seasonal variations in plant extracts, bacterial growth kinetics) leads to inconsistencies in nanoparticle size, shape, surface charge, and biological activity. Developing standardized green synthesis protocols and rigorous quality control measures is imperative.
- Scalability and Downstream Processing: Transitioning from small-scale laboratory green synthesis to large-scale industrial manufacturing remains challenging. Optimizing bioreactor parameters, extraction yields, and purification steps without compromising particle stability is a critical focus for bioengineers.
- Comprehensive *in vivo* Toxicity and Pharmacokinetics: While BNPs generally demonstrate lower toxicity and superior biocompatibility compared to chemically synthesized counterparts, comprehensive long-term ecotoxicological, pharmacokinetic, biodistribution, and clearance profiles are still needed to satisfy regulatory frameworks for clinical and agricultural use.

## 2 Conclusion

The articles compiled in this Research Topic collectively underscore the transformative potential of biogenic nanoparticles across biomedical, environmental, and agricultural biotechnology. By bridging molecular genetics, biological synthesis, advanced physical characterization, and translational microbiology, these contributions emphasize that biogenic nanomaterials are not merely “green” substitutes for synthetic particles, but advanced functional tools with tunable properties. Continued interdisciplinary collaboration will be crucial for accelerating the transition of BNP technologies from experimental platforms to standardized, real-world biotechnological solutions.
